# Macrophage SREBP1 regulates skeletal muscle regeneration

**DOI:** 10.3389/fimmu.2023.1251784

**Published:** 2024-01-08

**Authors:** Yumiko Oishi, Hiroyuki Koike, Naoki Kumagami, Yoshimi Nakagawa, Masaya Araki, Yoshitaka Taketomi, Yoshimi Miki, Shigeru Matsuda, Hyeree Kim, Takashi Matsuzaka, Hitoshi Ozawa, Hitoshi Shimano, Makoto Murakami, Ichiro Manabe

**Affiliations:** ^1^ Department of Medical Biochemistry, Graduate School of Medical and Dental Sciences, Tokyo Medical and Dental University, Tokyo, Japan; ^2^ Department of Biochemistry & Molecular Biology, Nippon Medical School, Tokyo, Japan; ^3^ Division of Complex Bioscience Research, Department of Research and Development, Institute of Natural Medicine, University of Toyama, Toyama, Japan; ^4^ Department of Endocrinology and Metabolism, Institute of Medicine, University of Tsukuba, Ibaraki, Japan; ^5^ Laboratory of Microenvironmental Metabolic Health Sciences, Center for Disease Biology and Integrative Medicine, Graduate School of Medicine, The University of Tokyo, Tokyo, Japan; ^6^ Department of Obstetrics and Gynecology, Nippon Medical School, Tokyo, Japan; ^7^ Department of Systems Medicine, Chiba University Graduate School of Medicine, Chiba, Japan; ^8^ Department of Anatomy and Neurobiology, Graduate School of Medicine, Nippon Medical School, Tokyo, Japan

**Keywords:** macrophage, SREBP (sterol regulatory element-binding protein) pathway, EPA - 20:5n-3, skeletal muscle regeneration, fatty acid metabolism

## Abstract

Macrophages are essential for the proper inflammatory and reparative processes that lead to regeneration of skeletal muscle after injury. Recent studies have demonstrated close links between the function of activated macrophages and their cellular metabolism. Sterol regulatory element-binding protein 1 (SREBP1) is a key regulator of lipid metabolism and has been shown to affect the activated states of macrophages. However, its role in tissue repair and regeneration is poorly understood. Here we show that systemic deletion of *Srebf1*, encoding SREBP1, or macrophage-specific deletion of *Srebf1a*, encoding SREBP1a, delays resolution of inflammation and impairs skeletal muscle regeneration after injury. *Srebf1* deficiency impairs mitochondrial function in macrophages and suppresses the accumulation of macrophages at sites of muscle injury. Lipidomic analyses showed the reduction of major phospholipid species in *Srebf1* ^-/-^ muscle myeloid cells. Moreover, diet supplementation with eicosapentaenoic acid restored the accumulation of macrophages and their mitochondrial gene expression and improved muscle regeneration. Collectively, our results demonstrate that SREBP1 in macrophages is essential for repair and regeneration of skeletal muscle after injury and suggest that SREBP1-mediated fatty acid metabolism and phospholipid remodeling are critical for proper macrophage function in tissue repair.

## Introduction

Macrophages are a diverse cell population that are essential for tissue repair and regeneration (1, 2). Skeletal muscle, for example, is a highly regenerative tissue, but its regeneration process is severely impaired when macrophages are depleted (3). After skeletal muscle is injured, circulating monocytes infiltrate the tissue at the site of injury and then within 1-2 days differentiate into pro-inflammatory macrophages that express a high level of the surface marker Ly6C (Ly6C^hi^). The Ly6C^hi^ macrophages not only clear muscle debris, they also activate satellite cells to initiate the regeneration process (4). Within 2-3 days after injury, the macrophage population at the site of injury becomes dominated by Ly6C^lo^ macrophages, which are characterized by high expression of CD163 and anti-inflammatory cytokines, such as IL-10. These Ly6C^lo^ macrophages are thought to contribute to muscle repair and regeneration (5, 6). Previous studies have shown that both early Ly6C^hi^ and late Ly6C^lo^ macrophages are required for muscle regeneration and that the proper transition of macrophages from the Ly6C^hi^ to Ly6C^lo^ phenotype is critical (7).

Recent studies have revealed the close mechanistic links between the functions of macrophages and their cellular metabolism. Activation of glycolysis is coupled to macrophage function during early inflammation (8). On the other hand, the resolution of inflammation is characterized by metabolic rewiring from glycolysis in pro-inflammatory macrophages towards oxidative phosphorylation in anti-inflammatory macrophages (9, 10). It has also been reported that efferocytosis is a major event that shifts the macrophage phenotype toward the resolution of inflammation necessary for skeletal muscle regeneration (11–13).

We previously reported that a sterol regulatory element-binding protein 1 (SREBP1)-mediated epigenetic regulatory mechanism coupled with cellular lipid metabolism plays a central role in the control of the transition of macrophages from the pro-inflammatory to pro-resolution phenotype (14). SREBP1 is a key transcription factor that regulates lipid metabolism. Among the three SREBP isoforms expressed in mammalian cells, SREBP1a and SREBP1c are both encoded by the *Srebf1* gene and are generated through alternative splicing, while SREBP2 is encoded by *Srebf2*. SREBP1a is the major isoform expressed in macrophages (15, 16). Cultured *Srebf1* ^-/-^ macrophages exhibit an enhanced and prolonged inflammatory response to TLR4 activation, while *Srebf1* ^-/-^ mice exhibit impaired inflammatory resolution in a lipopolysaccharide-mediated endotoxin shock model (14). Levels of unsaturated fatty acids are reduced in *Srebf1* ^-/-^ macrophages, and supplementation of exogenous eicosapentaenoic acid (EPA) prior to the lipopolysaccharide challenge protects *Srebf1^-/-^* mice from the otherwise exaggerated inflammatory response. Thus, SREBP1-dependent fatty acid metabolism appears to be critical for inflammatory resolution. In subsequent studies, we and others showed that during the response to lipopolysaccharide, SREBP1a is activated by caspase-11- and mTORC1-mediated pathways in macrophages (16–18). Although it is evident that immune cells adopt unique metabolic programs specific to their state and environment, it is unclear how SREBP1 regulates muscle regeneration and repair through regulation of macrophage function. In the present study, therefore, we investigated whether and how SREBP1-mediated metabolic alterations in macrophages affect muscle repair and regeneration after injury.

## Results

### Muscle repair and regeneration are impaired in *Srebf1^-/-^* mice

To begin addressing the possible role of SREBP1 in skeletal muscle regeneration and tissue repair, we evaluated the morphology of skeletal muscle in *Srebf1* ^-/-^ mice. While an earlier study showed that overexpression of SREBP1a or 1c in myoblasts inhibited differentiation into myotubes and induced muscle atrophy *in vivo* (19), we found no apparent differences in muscle histology between *Srebf1^-/-^* and wild-type (WT) mice in the steady state (**Supplementary Figure 1**).

**Figure 1:**
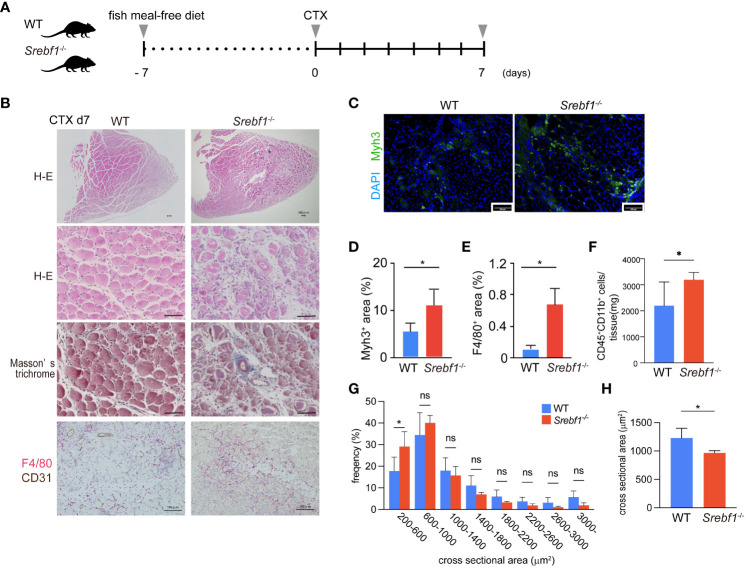
Delayed regeneration and prolonged inflammation after muscle injury in *Srebf1^-/-^* mice **(A)** Experimental scheme. *Srebf1^-/-^* and WT mice were fed a fish meal-free diet for 7 days, followed by injection of cardiotoxin to injure tibialis anterior muscles. **(B)** Representative H-E-stained sections of muscle collected 7 days after cardiotoxin injection. Tissue fibrosis was assessed by Masson-trichrome staining. F4/80- (red) and CD31-positive (brown) areas were visualized by immunostaining. Scale bars, 100 μm. **(C)** Representative photographs of injured muscle immunostained for Myh3. Scale bars, 100 μm. **(D, E)** Myh3- **(D)** or F4/80-positive **(E)** areas were quantified and normalized to the entire muscle section area. n=10 in each group. *P<0.05, Student’s two-tailed t test. **(F)** Flow cytometric analysis of cells collected from injured muscle 7 days post-injury. Numbers of CD45^+^ CD11b^+^myeloid cells/mg tissue are shown. **(G)** Distribution of muscle fiber cross-sectional areas. Shown are the mean numbers of muscle fibers within the indicated area ranges per 2400 myofibers/genotype. Data are means ± SD. *P < 0.05, one-way ANOVA and Tukey’s test for multiple comparisons. **(H)** Average muscle fiber cross-sectional area among 2400 myofibers/genotype counted. n=10 in each group. *P<0.05, Student’s two-tailed *t* test. ns= not significant.

To investigate whether SREBP1 contributes to the repair of skeletal muscle after injury, cardiotoxin was injected into the tibialis anterior muscles of WT and *Srebf1* ^-/-^ mice (**Figure 1A**). We previously found that SREBP1 drives fatty acid elongation and desaturation, which promotes synthesis of polyunsaturated fatty acids in macrophages (14). The normal mouse chow diet contains 4.6 g of fat per 100 g of chow, and the fatty acid composition is 50% polyunsaturated fatty acids mainly derived from fish meal, 29% monounsaturated fatty acids and 21% saturated fatty acids (**Supplementary Table 1**). Because of the high polyunsaturated fatty acid content derived from fish meal in the chow diet, we changed the diet to fish meal-free diet 7 days prior to the cardiotoxin injury so that phenotypes related to the generation of polyunsaturated fatty acids might be enhanced (**Figure 1A**).

Hematoxylin and eosin (H-E) staining of the injured muscle tissues in WT mice 7 days after injury revealed efficient muscle regeneration characterized by replacement of most necrotic fibers by small centrally-nucleated regenerating myofibers (**Figure 1B**). A minor portion of the myofibers modestly expressed Myh3, a marker of immature myofibers (**Figures 1C, D**), and numerous F4/80^+^ macrophages surrounded the regenerating myofibers (**Figure 1B**). By contrast, *Srebf1^-/-^* mice exhibited markedly impaired muscle regeneration with more cellular debris and many small regenerating myofibers that strongly stained for Myh3 within *Srebf1* ^-/-^ muscles (**Figures 1C, D**). Because Myh3 is a marker of immature fibers, its persistent expression is an indicator of delayed myotube maturation. In addition, there were many more F4/80^+^ macrophages within the injured *Srebf1^-/-^* muscles than within WT muscles (**Figures 1B, E**). We then used flow cytometry to further assess the numbers of CD45^+^ CD11b^+^ myeloid cells. As shown in **Figure 1F**, many more CD45^+^ CD11b^+^ cells persistently infiltrated the *Srebf1*-deficient muscle than the WT muscle. Taken together, these findings indicate that myotube maturation and regeneration is delayed in *Srebf1*-deficient muscle.

Consistent with that delay, the distribution of cross-sectional areas indicated that *Srebf1* deletion shifted regenerating myofibers towards smaller cross-sectional areas (**Figure 1G**) such that the average cross-sectional area was significantly smaller in *Srebf1^-/-^* than WT muscle (**Figure 1H**). By day 14 after injury, the skeletal muscle was repaired in both *Srebf1^-/-^* and WT mice. However, greater numbers of interstitial cells remained in the *Srebf1* ^-/-^ tissues (**Supplementary Figure 1**). Collectively, these results indicate impaired clearance of cell debris, prolonged inflammation with sustained macrophage accumulation, and delayed muscle regeneration and tissue repair in *Srebf1^-/-^* mice.

### *Srebf1* deficiency does not affect satellite cell differentiation

Because the delayed muscle repair in *Srebf1* ^-/-^ mice could be due to a differentiation defect in *Srebf1*-deficient satellite cells (19), we isolated satellite cells from tibialis anterior muscles from WT and *Srebf1^-/-^* mice and assessed their differentiation potentials. The isolated satellite cells had comparable morphologies, and the levels of Pax7 and MyoD1 expression were also comparable (**Supplementary Figures 2A, B**). When these cells were induced to differentiate, the mRNA expression levels of the myogenic transcription factors *Myf5*, *Myod1* and *Myog* and the myotube differentiation marker *Myh3* did not differ between the two genotypes on day 4 after differentiation (**Supplementary Figure 2C**). Moreover, *Srebf1^-/-^* satellite cells similarly differentiated into spindle-shaped myotubes during the 3-day culture period (**Supplementary Figure 2A**). These findings indicate that the lack of *Srebf1* in satellite cells does not affect their differentiation into myotubes, at least in this *in vitro* setting.

### Monocyte/macrophage recruitment is attenuated at the transition phase in *Srebf1^-/-^* muscle

**Figure 2:**
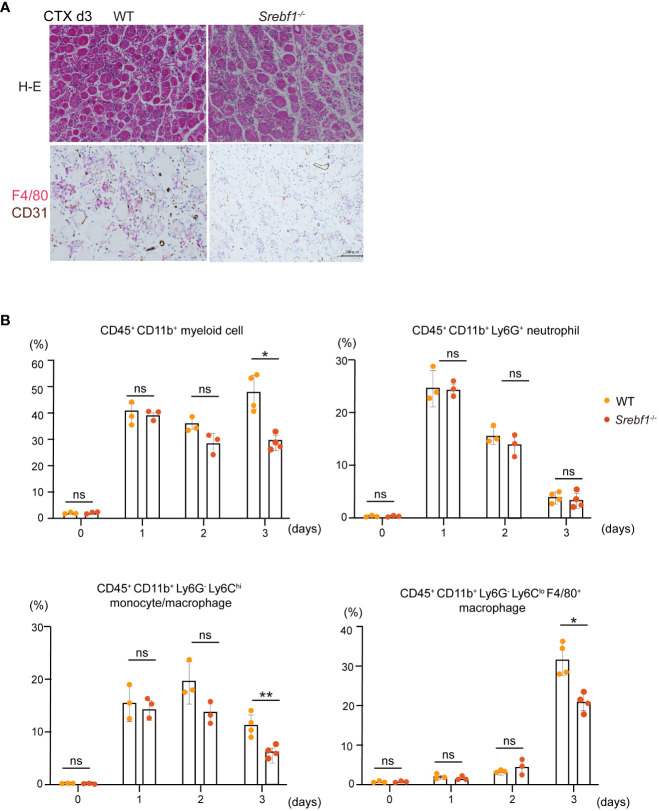
Macrophage recruitment to injury sites was attenuated 3 days post-injury in *Srebf1^-/-^* mice **(A)** Representative H-E-stained sections of muscle collected 3 days after cardiotoxin injection. F4/80- (red) and CD31-positive (brown) areas were visualized by immunostaining. Scale bars, 100 μm. **(B)** Flow cytometric analysis of cells collected from injured muscle on the indicated days post-injury. Shown are the proportions of CD45^+^ CD11b^+^myeloid cells, CD45^+^ CD11b^+^ Ly6G^+^ neutrophils, CD45^+^ CD11b^+^ Ly6C^+^ monocytes, CD45^+^ CD11b^+^ Ly6C^-^ F4/80^+^ macrophages. n=3-4 mice in each group. *P <0.05, Student’s 2-tailed t test. **P<0.01, ns= not significant.

We next analyzed the temporal changes in myeloid cells early during the injury and repair process (i.e., days 1 to 3) after cardiotoxin injection. Massive myofiber necrosis and scattered inflammatory cells were observed on day 1 post-injury, and the numbers of infiltrating cells increased to day 3, as reported previously (4) (**Supplementary Figure 1**). Although the numbers of infiltrating myeloid cells were comparable between WT and *Srebf1* ^-/-^ muscles on days 1 and 2 after injury, by day 3 accumulation of F4/80^+^ macrophages within the injured tissues was less in *Srebf1^-/-^* than WT mice (**Figure 2A**; **Supplementary Figure 1**).

Flow cytometrical analyses showed that while the CD45^+^ CD11b^+^ myeloid cell fractions did not differ between WT and *Srebf1^-/-^* muscles on days 1 and 2, there were significantly fewer myeloid cells in *Srebf1^-/-^* muscles on day 3 (**Figure 2B**). Among the myeloid cells, the Ly6G^+^ neutrophil fraction was comparable between WT and *Srebf1* ^-/-^ muscles. However, the Ly6C^hi^ monocyte/macrophage fractions tended to be smaller in *Srebf1* ^-/-^ muscles on day 2 and were significantly reduced on day 3 post-injury as compared with WT muscles. Likewise, numbers of Ly6C^lo^F4/80^+^ macrophages were also smaller in *Srebf1* ^-/-^ muscles than WT muscles on day 3 post-injury (**Figure 2B**).

### Myeloid *Srebf1* is required for repair and regeneration after cardiotoxin-induced muscle injury

**Figure 3:**
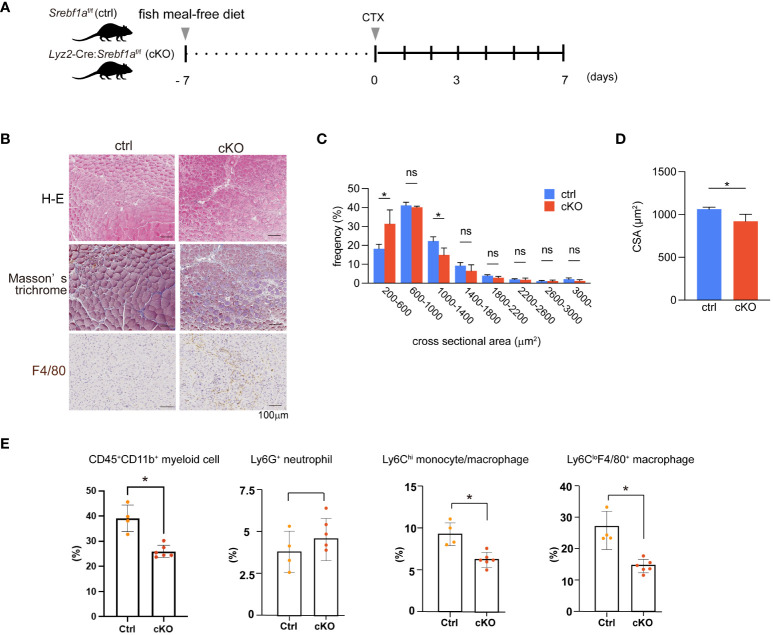
Delayed regeneration and prolonged inflammation in macrophage-selective SREBP1a-deleted mice **(A)** Experimental scheme. *Lyz2*-Cre : *Srebf1a* ^f/f^ and *Srebf1a* ^f/f^ control mice were fed a fish meal-free diet for 7 days, followed by cardiotoxin injection to injure the tibialis anterior muscles. **(B)** Representative H-E-stained sections of muscle collected 7 days after cardiotoxin injection. Tissue fibrosis was assessed by Masson-trichrome staining. F4/80-positive (brown) areas were visualized by immunostaining. Scale bars, 100 μm. **(C)** Distribution of muscle fiber cross-sectional areas. Data are means ± SD. Shown are the mean numbers of muscle fibers within the indicated area ranges per 2,400 myofibers/genotype. *P < 0.05, one-way ANOVA and Tukey’s test for multiple comparisons. **(D)** Average muscle fiber cross-sectional area among 2400 myofibers/genotype counted. n=10 in each group. *P<0.05, Student’s two-tailed *t* test. **(E)** Flow cytometric analysis of cells from injured muscle collected on day 3 post-injury. Shown are the proportions of CD45^+^ CD11b^+^ myeloid cells, CD45^+^ CD11b^+^ Ly6G^+^ neutrophils, CD45^+^ CD11b^+^ Ly6G^-^Ly6C^+^moyocyte, and CD45^+^ CD11b^+^ Ly6C^-^ F4/80^+^ macrophages. n = 4-6 mice in each group. *P <0.05, Student’s 2-tailed t test. ns= not significant.

The results so far suggest that changes in monocytes and macrophages may contribute to the prolonged inflammation and delayed muscle regeneration seen in *Srebf1^-/-^* mice. To further address that, we established a myeloid cell-specific *Srebf1a* knockout mouse (*Lyz2*-Cre: *Srebf1a* ^f/f^, cKO) by crossing mice carrying the floxed *Srebf1a* allele (20) and *Lyz2*-driven Cre (21). In the steady state, cKO mice developed normally with no discernable differences in skeletal muscle morphology. After 7 days on a fish meal-free diet, these mice were subjected to cardiotoxin-mediated muscle injury (**Figure 3A**). In the control *Srebf1a* ^f/f^ mice, the injured muscle tissues were mostly repaired and regenerated by day 7 post-injury, with newly generated and arranged myofibers containing central nuclei, as we observed in WT mice (**Figures 1B**, **3B**). Moreover, there were no differences in the distribution of muscle fiber cross-sectional areas between WT and *Srebf1a* ^f/f^ mice (**Supplementary Figure 3**). By contrast, extensive tissue damage was still apparent within cKO muscles. Large numbers of cells and much deposition of extracellular matrix (ECM) was observed within the interstitium of the injured cKO tissues (**Figure 3B**). Moreover, numerous necrotic myofibers that stained blue with Masson’s trichrome stain remained (**Figure 3B**). The majority of interstitial cells stained positively for F4/80, indicating that macrophage accumulation was sustained on day 7. There were more small fibers in cKO than control mice (**Figure 3C**), and the average cross-sectional area was smaller in cKO than control muscles (**Figure 3D**), suggesting delayed maturation of the myofibers. Thus, cKO mice phenocopied the systemic *Srebf1*-deficient mice in the cardiotoxin muscle injury model and exhibited prolonged macrophage accumulation, impaired clearance of dead myocytes, and impaired regeneration on day 7 post-injury. These findings support the idea that the lack of *Srebf1a* in myeloid cells, but not satellite cells, impairs muscle repair and regeneration.

To further assess the dynamics of myeloid cells during the course of repair and regeneration, we performed flow cytometric analyses of muscle stromal cells collected from both genotypes. The fractions of CD45^+^ CD11b^+^ myeloid cells, Ly6C^hi^ monocytes/macrophages and Ly6C^lo^F4/80^+^ macrophages were all significantly smaller in cKO muscles than control muscles on day 3 post-injury, whereas the Ly6G^+^ neutrophil fractions did not differ (**Figure 3E**), as was observed in *Srebf1* ^-/-^ muscles (**Figure 2B**). These findings demonstrate that the lack of *Srebf1a* in myeloid cells was sufficient to reduce macrophage accumulation on day 3 post-injury and suggest that this defect in macrophage accumulation likely contributes to the impaired repair and regeneration in cKO muscles.

### *Cx3cr1* is downregulated in macrophages in cKO mice

**Figure 4:**
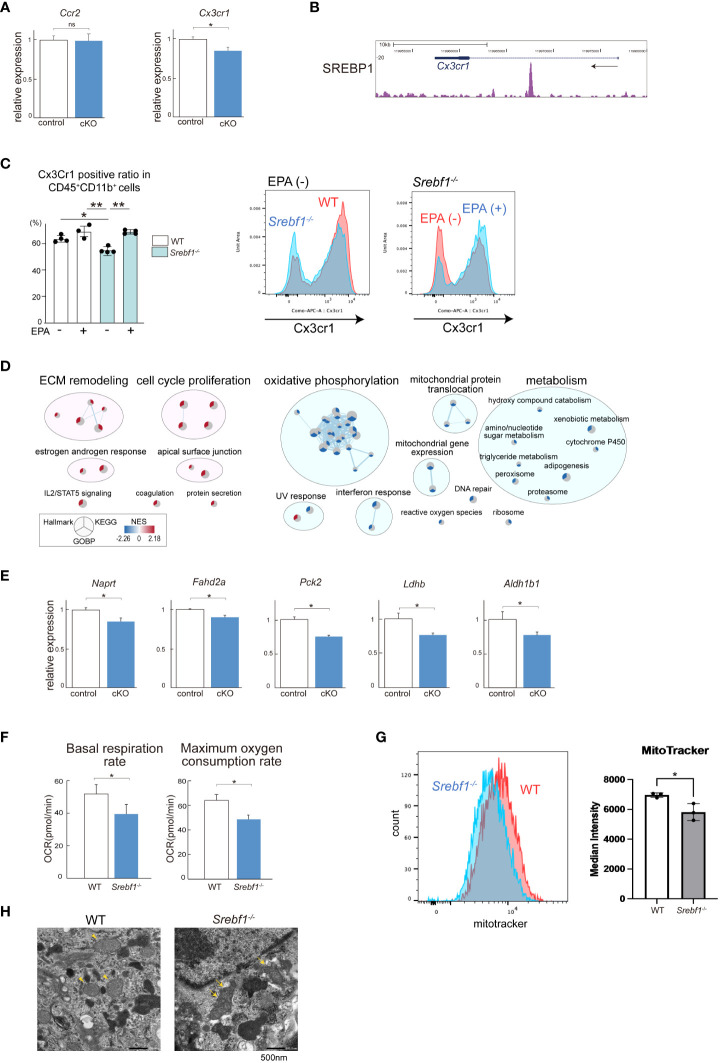
SREBP1a deletion modulates mitochondrial function and CX3CR1 expression **(A)** Relative levels of *Ccr2* and *Cx3cr1* mRNA expression in CD45^+^ CD11b^+^ myeloid cells from injured muscle collected 3 days after cardiotoxin injection. *P < 0.05, Student’s 2-tailed t test. Data are shown as the mean ± SD. **(B)** Gene locus of mouse *Cx3cr1*. The UCSC Genome Browser shot shows the ChIP-seq result for SREBP1 in mouse BMDMs. **(C)** Proportion of Cx3Cr1-positive cells in the myeloid cell population quantified by flow cytometric analysis. Analyzed were the CD45^+^ CD11b^+^ population obtained from bone marrow collected from mice fed with control or EPA-supplemented diet for 7 days. n = 3-4 mice for each group. *P <0.05, Tukey-Kramer *post hoc* test. **(D)** RNA-Seq results for control (*Srebf1a* ^f/f^) and *Lyz2*-Cre: *Srebf1a* ^f/f^ CD45^+^ CD11b^+^ myeloid cells collected from injured muscle 3 days post-injury. Differentially expressed gene sets (FDR < 0.1) from the MSigDB hallmark, KEGG, and GO biological process databases were clustered using EnrichmentMap and AutoAnnotate (26). Further details are shown in **Supplementary Table 2**. **(E)** Relative levels of mRNA expression of mitochondria-related genes in CD45^+^ CD11b^+^ myeloid cells from injured muscle. *P < 0.05, Student’s two-tailed t test. Data are shown as means ± SD in all panels where P values are shown. **(F, G)** BMDMs were obtained from *Srebf1^-/-^* and WT mice. The basal respiration and maximum oxygen consumption rates were analyzed using a flux analyzer **(F)**. Mitochondrial membrane potential was assessed by staining with MitoTracker Red and analyzed with flow cytometry **(G)**. **(H)** Transmission electron microscopic analysis of injured tibialis anterior muscle collected 3 days post-injury. Representative images from four independent injured muscles are shown. The arrowheads point to mitochondria with normal structures. The arrows point to degenerative changes. Magnification ×10,000. Scale bar: 500nm.

To begin to determine how the loss of *Srebf1* alters the macrophage response to muscle injury, we first addressed how monocyte/macrophage accumulation was suppressed in cKO mice. Earlier studies showed that inflammatory monocytes recruited from the circulation are characterized by a Ly6C^hi^Cx3cr1^lo^ surface phenotype and that their recruitment is CCR2-dependent (4). The Ly6C^hi^Cx3cr1^lo^ monocytes then switched their phenotype to Ly6C^lo^ macrophages within the muscle, and a large portion of these cells also exhibited Cx3cr1^hi^ (4). Similarly, expression of Cx3cr1 also marks the transition of monocytes/macrophages from the acute inflammation phase to the resolution/repair phase (22–25). In cKO myeloid cells within injured muscles, *Ccr2* expression was comparable to that in WT muscles, but *Cx3cr1* expression was significantly decreased (**Figure 4A**). Our earlier ChIP-seq (GSE79423) data showed that SREBP1 binds to the *Cx3cr1* gene locus, which suggests SREBP1 directly regulates *Cx3cr1* expression (**Figure 4B**). To determine whether Cx3cr1 expression is decreased in *Srebf1*-deficient mice, the proportion of Cx3cr1^+^ cells in the bone marrow was analyzed. The result confirmed that the proportion of Cx3cr1-positive cells among CD45^+^CD11b^+^ cells was significantly smaller in *Srebf1^-/-^* than WT mice (**Figure 4C**).

### SREBP1 is essential for normal mitochondrial function in macrophages

In addition to the impaired accumulation of monocytes/macrophages, it is likely that the lack of *Srebf1a* also modulates macrophage function within the injured tissues (14). To address this, we isolated CD45^+^ CD11b^+^ myeloid cells from injured muscle collected from cKO and control mice on day 3 post-injury and analyzed their transcriptomes using RNA-seq. At this time point, the majority (>90%) of CD45^+^ CD11b^+^ myeloid cells were macrophages or monocytes (**Figure 3E**). In the *Srebf1a*-deficient myeloid cells, the gene sets related to ECM signaling and tissue remodeling, such as TGF-β signaling and epithelial-mesenchymal transition, were upregulated (**Figure 4D**; **Supplementary Table 2**).

Numerous gene sets related to cellular metabolism were downregulated in *Srebf1a*-deficient cells. In particular, a large cluster of the gene sets related to mitochondrial oxidation and electron transport as well as those related to mitochondrial gene and protein expression were downregulated in *Srebf1a*-deficient cells, suggesting an impact on mitochondrial function (**Figure 4D**). qPCR analysis confirmed that expression of genes encoding mitochondrial enzymes, including *Naprt*, *Fahd2a*, *Pck2*, *Ldhb* and *Aldh1b1*, was reduced in cKO myeloid cells within the injured muscle (**Figure 4E**). Our earlier ChIP-seq (GSE79423) data showed that SREBP1 binds to the *Naprt*, *Fahd2a*, *Pck2*, *Ldhb* and *Aldh1b1* gene loci, which suggests SREBP1 directly regulates expression of those genes (**Supplementary Figure 4**). We therefore hypothesized that mitochondrial activity is altered by *Srebf1* deletion in macrophages. To test that idea, we used a flux analyzer to measure mitochondrial respiration in bone marrow-derived macrophages (BMDMs). We found that the basal oxygen consumption rate and maximum oxygen consumption rate were both significantly decreased in *Srebf1^-/-^* BMDMs (**Figure 4F**).

Mitochondrial membrane potential was also significantly decreased in *Srebf1^-/-^* BMDMs (**Figure 4G**). High-resolution scanning electron microscopy revealed the presence of swollen and whirled cristae, consistent with mitochondrial damage, in macrophage mitochondria from *Srebf1^-/-^* muscles on day 3 post-injury (**Figure 4H**). *Srebf1* thus appears to be important for maintaining mitochondrial homeostasis in macrophages.

### *Srebf1* deficiency reduces levels of major phospholipids in myeloid cells in injured muscles

**Figure 5:**
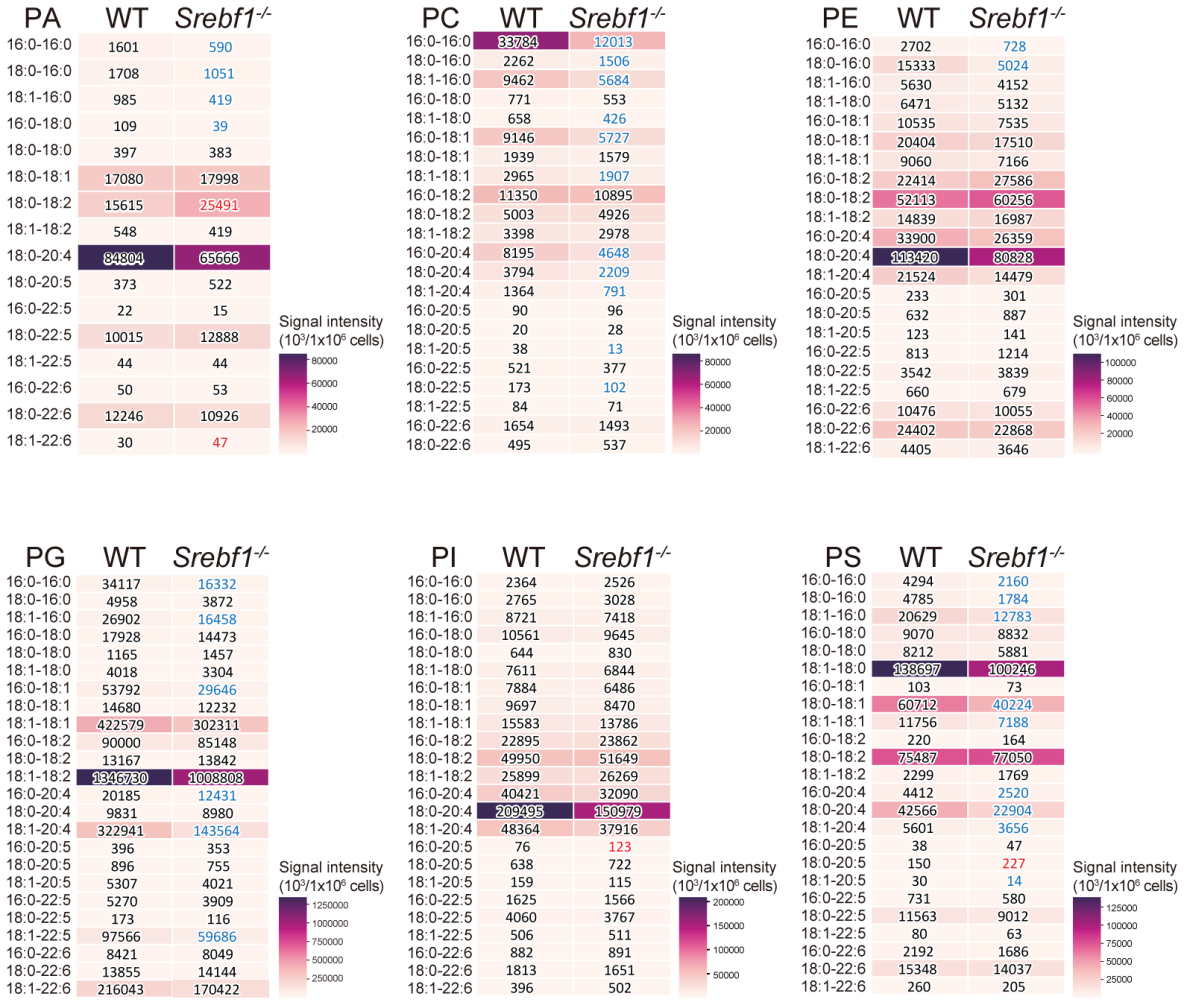
SREBP1 deletion caused reduction of major phospholipid species PA, PC, PE, PG, PI and PS profiles were analyzed using liquid chromatography-MS. Analyzed were CD45^+^ CD11b^+^ myeloid cells collected from muscle 3 days post-injury. Heatmaps show mean signal intensities of phospholipid groups in WT and *Srebf1^-/-^* cells (n=4). Signal intensity values are also shown. *Srebf1^-/-^* cell intensity values that are more than 1.5-fold higher or lower than WT cells are shown in red and blue, respectively.

So far, we have shown that *Srebf1* deletion impairs mitochondrial function and alters expression of genes involved in ECM signaling and tissue remodeling in macrophages. Because we previously observed that SREBP1 drives fatty acid desaturation and elongation in macrophages *in vitro* (14), we hypothesized that altered lipid metabolism may contribute to the modulated function of *Srebf1* ^-/-^ macrophages. To test that idea, we first performed a lipidomics analysis to detect the phospholipids in sorted myeloid cells from injured muscle on day 3 post-injury. Loss of *Srebf1* resulted in decrease in the levels of major phospholipids. In particular, phospholipids containing C20:4 that consists of eicosatetraenoic acid (n-3) or arachidonic acid (n-6) as sn-2 fatty acids were decreased in *Srebf1^-/-^* cells (**Figure 5**).

### EPA supplementation promotes muscle regeneration in *Srebf1^-/-^* mice

**Figure 6:**
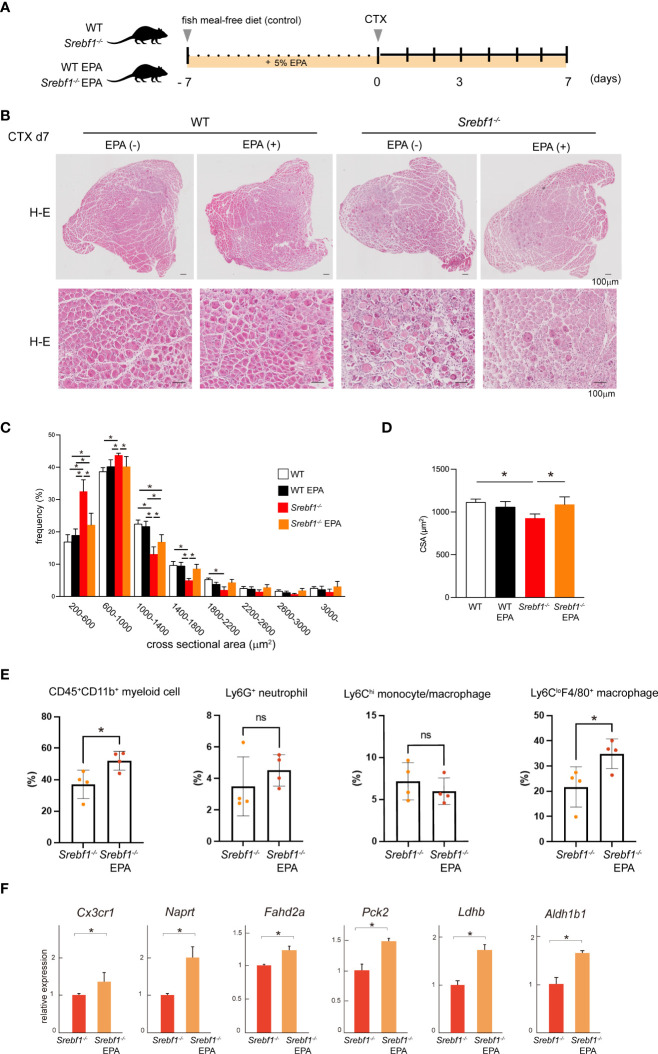
EPA supplementation ameliorates the delayed muscle regeneration in *Srebf1^-/-^* mice **(A)** Experimental scheme. WT and *Srebf1^-/-^* mice were fed a fish meal-free diet or EPA-rich diet for 7 days, followed by the cardiotoxin injection to damage the tibialis anterior muscles. **(B)** Representative H-E-stained sections of muscle collected 7 days after cardiotoxin injection into WT and *Srebf1^-/-^* mice. Upper panel: low magnification picture. Lower panel: high magnification picture. Scale bars: 100 μm. **(C)** Distribution of muscle fiber cross-sectional areas. Data are means ± SD. Shown are the mean numbers of muscle fibers within the indicated area ranges per 2,400 myofibers/experimental condition. *P < 0.05, one-way ANOVA and Tukey’s test for multiple comparisons. Only significant comparisons are shown. **(D)** Average muscle fiber cross-sectional area among 2,400 myofibers/condition counted. n=10 in each group. *P < 0.05, one-way ANOVA and Tukey’s test for multiple comparisons. Only significant comparisons are shown. **(E)** Flow cytometric analysis of cells from injured muscle collected on day 3 post-injury. Shown are the proportions of CD45^+^ CD11b^+^ myeloid cells, CD45^+^ CD11b^+^ Ly6G^+^ neutrophils, CD45^+^ CD11b^+^ Ly6C^+^ F4/80^+^ monocyte/macrophages, and CD45^+^ CD11b^+^ Ly6C^-^F4/80^-^ cells. n = 6-7 mice in each group. *P <0.05, Student’s 2-tailed t test. **(F)** Relative mRNA expression of *Cxc3cr1* and mitochondria-related genes in CD45^+^ CD11b^+^ myeloid cells from injured muscle. *P < 0.05, Student’s two-tailed t test. Data are shown as means ± SD in all panels where P values are shown.

We previously reported that *Srebf1*-deficient mice showed enhanced and prolonged secretion of pro-inflammatory cytokines after systemic LPS challenge. However, the prolonged inflammatory response was abrogated by supplementation with EPA (14). To assess whether abnormal phospholipid composition in macrophages contributes to the delayed response to muscle injury in *Srebf1*-deficient mice, we analyzed the effects of supplementing the diet of WT and *Srebf1* ^-/-^ mice with EPA. The mice were fed a control (fish meal-free) diet or EPA-rich diet for 7 days prior to muscle injury (**Figure 6A**). Seven days after cardiotoxin injection, we did not detect apparent histological differences between WT mice fed the control or EPA-rich diet. In the *Srebf1* ^-/-^ mice fed the control diet, inflammation characterized by the presence of numerous interstitial cells persisted and necrotic fibers and immature myofibers were also still observed (**Figure 6B**), as is also seen in **Figure 1B**. In sharp contrast, much fewer infiltrating interstitial cells were observed in the *Srebf1* ^-/-^ mice fed the EPA-rich diet. Moreover, muscle fiber regeneration and maturation appeared to be promoted (**Figure 6B**). Consistent with those observations, the regenerating myofibers were shifted toward larger cross-sectional areas in *Srebf1* ^-/-^ mice fed the EPA-rich diet, whereas EPA supplementation did not affect fiber cross-sectional areas in WT mice (**Figures 6C, D**).

We then analyzed whether EPA supplementation would restore monocyte/macrophage recruitment by flow cytometry. We observed that in mice fed the EPA diet, the CD45^+^CD11b^+^ myeloid cell fraction was significantly increased on day 3 post-injury (**Figure 6E**). And while the Ly6C^hi^ monocyte/macrophage fractions were unaffected, numbers of Ly6C^lo^F4/80^+^ macrophages were increased by EPA supplementation. EPA supplementation also increased expression of both Cx3cr1 mRNA in CD45^+^CD11b^+^ myeloid cells collected from the injury site (**Figure 6E**) and the proportion of Cx3cr1-positive cells among CD45^+^CD11b^+^ cells in bone marrow (**Figure 4C**). In addition, qPCR analysis of mRNA expression in CD45^+^ CD11b^+^ myeloid cells collected from injured muscle on day 3 post-injury showed that EPA supplementation increased expression of the mitochondrial enzyme genes *Naprt*, *Fahd2a*, *Pck2*, *Ldhb* and *Aldh1b1* (**Figure 6F**). Taken together, these results suggest EPA supplementation restores the muscle tissue and monocyte/macrophage responses after muscle injury in *Srebf1* ^-/-^ mice.

**Figure 7:**
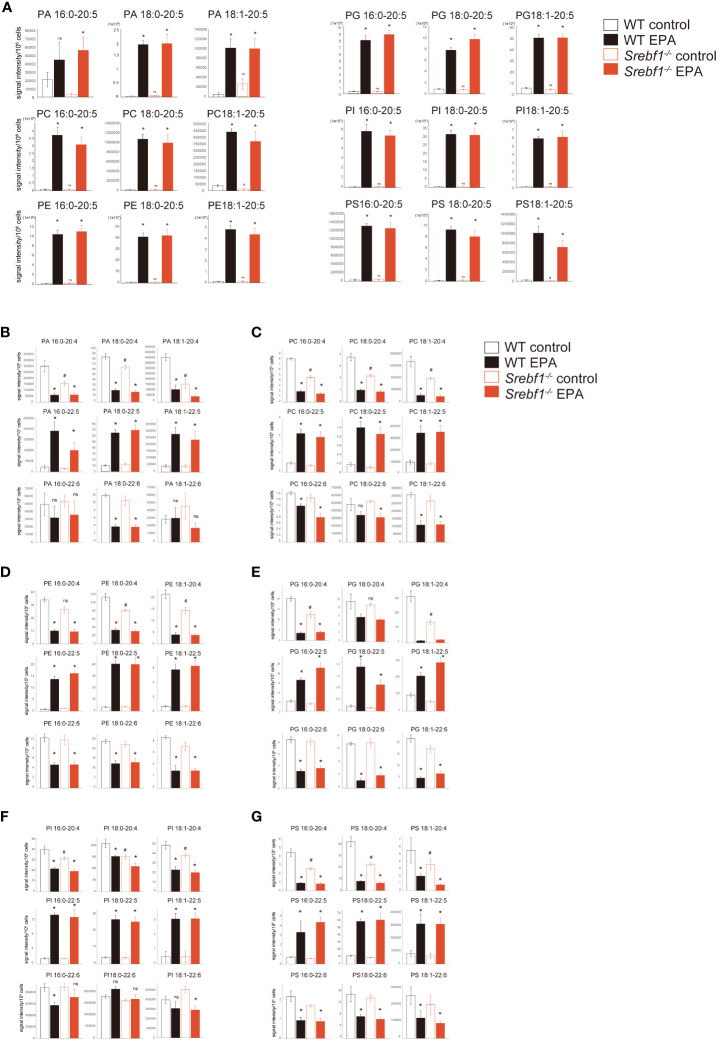
Phospholipid remodeling caused by the exogenous EPA supplementation CD45^+^ CD11b^+^ myeloid cells collected from muscle 3 days post-injury and analyzed using liquid chromatography-MS. **(A)** PA, PC, PE, PG, PI and PS containing EPA (C20:5) as sn-2 fatty acid. **(B–G)** PA **(B)**, PC **(C)**, PE **(D)**, PG **(E)**, PI **(F)** and PS **(G)** containing C20:4, C22:5 and C22:6 as sn-2 fatty acid. Data are shown as means ± SE in all panels where P values are shown. n =4 for each group. *P <0.05 compared to the same genotype fed with control diet, ^#^P <0.05 compared to the WT fed with control diet. ns; not significant. Tukey-Kramer *post hoc* test. Some of the data for the control diet are identical to those shown in **Figure 5**.

Finally, we determined whether an EPA-rich diet would alter the fatty acid composition of phospholipids. CD45^+^CD11b^+^ myeloid cells isolated from muscle tissue on day 3 after injury were subjected to lipidomics analysis. EPA supplementation increased the amount of EPA (C20:5)-containing phospholipid species in *Srebf1^-/-^* myeloid cells, with their levels reaching at least the same level detected in cells collected from WT mice fed a control diet (**Figure 7A**). Phospholipid species containing docosapentaenoic acid (C22:5) were also increased, while those containing C20:4 (eicosatetraenoic acid or arachidonic acid) were decreased (**Figures 7B–G**), suggesting possible phospholipid remodeling induced by EPA supplementation. In contrast, the levels of phospholipid species containing docosahexaenoic acid (C22:6) were not increased (**Figures 7B–G**).

For comparison, we also carried out EPA supplementation and lipidomics analysis in WT mice. Phospholipids containing EPA (C20:5) and docosapentaenoic acid (C22:5) were also increased and those containing C20:4 were decreased in WT mice fed an EPA-rich diet (**Figure 7**).

## Discussion

In the present study, we showed that systemic deletion of *Srebf1* and myeloid cell-specific deletion of *Srebf1a* prolongs inflammation and impairs regeneration after muscle injury. As reported in earlier studies, initially accumulated monocytes/macrophages were characterized by a Ly6C^hi^ phenotype, but around day 3 post-injury, Ly6C^lo^ macrophages became predominant within the injured tissues (4). Systemic deletion of *Srebf1* or myeloid-specific deletion of *Srebf1a* reduced the number of Ly6C^hi^ and Ly6C^lo^ macrophages present on day 3 (**Figures 2B**, **3E**). It is likely that the reduced accumulation of macrophages contributed to the impaired muscle regeneration seen in systemic *Srebf1*-deficient and myeloid-specific *Srebf1a*-deficient (cKO) mice. In addition to the reduced macrophage accumulation, altered macrophage function due to transcriptomic changes and mitochondrial dysfunction (**Figures 4D–H**) also likely contribute.

Our global transcriptome analyses demonstrated that gene sets related to mitochondrial metabolism are widely downregulated in SREBP1 deficient myeloid cells on day 3 after muscle injury (**Figure 4D**). The transcriptomic changes during the transition from initial inflammation to resolution/repair are characterized by downregulation of genes associated with glycolysis and upregulation of genes associated with elements of oxidative metabolism, including oxidative phosphorylation and fatty acid and glutamine metabolism (7). This suggests mitochondrial dysfunction affects the phenotype and function of macrophages during the transitional phase in *Srebf1*-deficient mice. Earlier studies showed that AMPK, which enhances fatty acid oxidation, is involved in the macrophage phenotype (27). Deletion of *Ampk1a* impairs the transition to the Ly6C^lo^ phenotype, decreases the numbers of accumulated Ly6C^lo^ macrophages on days 2 and 3 after muscle injury, and delays muscle regeneration (27). Phagocytosis of dead cells has been shown to be important for the macrophage phenotypic change by activating AMPK and fueling fatty acid oxidation (27, 28). Moreover, mitochondrial fission was shown to be required for the clearance of apoptotic cells by macrophages in atherosclerosis (29). These findings point to the essential roles of mitochondria in the macrophage actions toward inflammatory resolution and muscle regeneration.

Mitochondrial function in macrophages has also been shown to crucially impact resolution of inflammation and repair after myocardial infarction (30–32). Cai et al. reported that mitochondrial dysfunction in macrophages, induced by deletion of *Ndufs4*, which encodes NADH dehydrogenase, one of the components of the mitochondrial membrane respiratory chain, increased cell death among infiltrated macrophages and decreased numbers of both Ly6C^hi^ and reparative CD206^+^ macrophages on days 3 and 7 after myocardial infarction (30). *Ndufs4* ^-/-^ BMDMs showed impaired efferocytosis and blunted expression of reparative genes normally induced by efferocytosis of apoptotic cells (30). Although we need to further characterize the functional changes in *Srebf1* ^-/-^ macrophages within injured muscle, similar changes, including prolonged inflammation and decreased numbers of reparative macrophages, suggest that mitochondrial dysfunction in macropahges pivotally contributes to the impairment of macrophage accumulation and tissue repair after muscle injury in *Srebf1*-deficient mice.

It is not immediately clear why mitochondrial function is impaired in SREBP1-deficient macrophages. However, a global reduction of phospholipids (**Figure 5**) may account for the impaired mitochondrial function in SREBP1-deficient macrophages, as EPA-containing phospholipids are reportedly important for mitochondrial homeostasis in many cell types, including macrophages (33, 34). In agreement with this, exogenous EPA increased levels of phospholipids, including phosphatidylglycerol (PG) species containing EPA at their sn-2 position EPA (**Figure 7**), and increased expression of genes related to mitochondrial metabolism (**Figure 6F**). Because cardiolipin, which has been postulated to be important for mitochondrial function, is biosynthesized from PGs (35, 36), it seems likely that the phospholipid remodeling triggered by EPA supplementation plays a key role in the improved mitochondrial function. In addition, metabolites of EPA, such as resolvins, may also contribute to the effect of EPA on mitochondrial metabolism. For instance, Hosseini et al. reported that exogenous resolvin D1 enhances fatty acid oxidation and oxidative phosphorylation in macrophages, presumably via activation of AMPK signaling (37). Given that activation of AMPK is involved in the acquisition of the macrophage phenotype (27), our finding that EPA supplementation increased the numbers of Ly6C^lo^ macrophages after injury also suggests the involvement of AMPK signaling. On the other hand, AMPK has been shown to inhibit SREBP1 activity (38). Clearly it will be important to further delineate the interconnections among SREBP1, AMPK signaling and lipid metabolism in macrophages during inflammatory resolution and repair.

*Cx3Cr1* is one of the SREBP1 target genes in macrophages (**Figure 4B**). Consistent with that, Cx3cr1 protein expression was significantly downregulated in *Srebf1*-deficient monocytes/macrophages (**Figure 4C**). A large fraction of the Ly6C^lo^ macrophages that accumulate within injured muscle tissues express Cx3cr1 (4). Although Cx3cr1 has been shown to be important for the recruitment of monocytes to several inflammatory tissues, including myocardial infarction and atherosclerosis (31, 39), two previous studies also showed that Cx3cr1 is dispensable for the recruitment of Ly6C^lo^ macrophages to injured muscle tissues (23, 24). It therefore seems unlikely that the reduced accumulation of Ly6C^lo^ macrophages within *Srebf1* ^-/-^ muscles was due to the reduced expression of Cx3cr1. In addition to monocyte recruitment, Cx3cr1 signaling has also been shown to control macrophage survival, proliferation, polarization and metabolism in other tissues (31, 40–42). However, the role of Cx3cr1 signaling in muscle repair after injury remains unclear (23, 24). Interestingly, Lauro et al. showed that CX3CL1 promotes oxidative metabolism in both cultured microglia and microglia in the ischemic brain (42), suggesting that the reduced Cx3cr1 signaling might contribute to the metabolic dysfunction. In addition, although *Cx3cr1* is a target of SREBP1, EPA supplementation increased numbers of Cx3Cr1^+^ cells among bone marrow myeloid cells in *Srebf1* ^-/-^ mice. This indicates EPA can increase *Cx3cr1* expression independently of SREBP1. In the *in vitro* differentiation model, the lack of *Srebf1* did not affect differentiation of primary satellite cells into myotubes (**Supplementary Figure 2**). Because the culture medium contained mono- and polyunsaturated fatty acids, these lipids may have compensated for the loss of SREBP1. Further study is clearly needed to elucidate how EPA rescues cells from the loss of SREBP1.

In sum, our results demonstrate that SREBP1a in macrophages is important for muscle repair and regeneration after injury and suggest that SREBP1a-mediated regulation of lipid metabolism contributes to the phenotypic transition and reparative actions of macrophages within injured muscle.

## Materials and methods

### Reagents

Cardiotoxin was purchased from Sigma-Aldrich (C9759). EPA ethyl ester (97%) was generously provided from Bizen Chemical Co. Ltd (Okayama, Japan). Antibodies used in this study are listed in **Supplementary Table 3**.

### Mice

All mice used in this study had a C57BL/6 background. *Srebf1^-/-^* and *Srebf1* ^f/f^ mice were generated as described previously (20, 43). Male, 8- to 10-week-old *Srebf1* ^-/-^ and age-matched WT mice or *Lyz2*-Cre : *Srebf1a* ^f/f^ *^-^* and *Srebf1a* ^f/f^ *^-^* mice (as controls) were individually housed in cages in a 12h/12h light/dark cycle with free access to food and water. For studies involving supplemental EPA supplementation, mice were fed a fish meal-free diet (fish meal-free MF-1: 4.4% fat; Funabashi Farm) or fish meal-free diet supplemented with 5% EPA ethyl ester (v/v) for 7 days before cardiotoxin injection. All animal experiments were conducted after approval by the Institutional Animal Care Committee at the Nippon Medical School.

### Cardiotoxin-induced muscle regeneration

Male, 8- to 10-week-old mice were used for cardiotoxin injection experiments. Cardiotoxin was diluted to a final concentration of 10 μM in phosphate-buffered saline (PBS) and injected intramuscularly into the tibialis anterior muscles in a 50-μl volume. Muscles were then collected on the indicted days after cardiotoxin injection and snap frozen in liquid nitrogen-cooled isopentane. Mock-injected muscles from control animals were harvested at the same times.

### Histological analysis

Muscles were dissected from tendon to tendon, mounted on OCT, snap frozen in liquid nitrogen-cooled isopentane, cross-sectioned at 8-μm thickness on a cryostat and then mounted on slides for staining. For immunostaining, sections were first blocked in 3 mg/ml bovine serum albumin (BSA) in PBS then incubated overnight at 4°C with primary anti-Myh3 antibody diluted to 3 mg/ml in BSA/PBS. After washing in PBS, the sections were stained for 1 h with a secondary antibody added at a 1:250 dilution. After washing, the slides were counterstained with 4’,6-diamidino-2-phenylindole (DAPI) and visualized on a Zeiss fluorescence microscope using fluorescein-specific optics. For H-E and Masson-trichrome staining as well as F4/80 and CD31 immunostaining, muscle tissue was first fixed in methanol-added 18.5% formalin, embedding in paraffin and cut into 8-μm cross sections.

### Quantification of muscle morphometry

Cross-sectional area of myofibers were measured using CellSence software (Olympus) as described previously (44, 45). H-E images were photographed at ×20 magnification for measurements. Muscle fiber cross-sectional areas were measured from at least four different sections per condition, and histograms were prepared. The average of these measurements from all animals at each time point or condition was then used to determine the mean cross-sectional area. For cardiotoxin experiments comparing *Srebf1^-/-^* or *Lyz2*-Cre : *Srebf1a* ^f/f^ and control muscles, six to ten muscles with a total of 2,400 myofibers per condition were measured.

### Fluorescence activated cell sorting

After being fed a diet of fishmeal-free MF-1 for 7 days, mice were anesthetized with 1.5-2.0% isoflurane, and their tibialis anterior muscles were injected with 100 μl of PBS with or without 10 μM cardiotoxin (Sigma-Aldrich, St. Louis, MO, USA) using a 29-G syringe. The injected muscles were subsequently collected, minced, and enzymatically digested with 2 μg/ml collagenase II (Worthington Biochemical, Lakewood, NJ, USA) in HBSS (Nacalai Tesque, Japan) for 1 h at 37°C while shaking. The resultant cell suspensions were washed and filtered through 100-μm and then 40-μm cell strainers.

Monocytes were isolated from mouse bone marrow collected by perfusing the medullary cavities of femurs and tibias and suspended in a red blood cell lysis buffer. After lysing the red cells, the bone marrow cells were washed and filtered through a 40-μm cell strainer.

Pelleted muscle cells or monocytes were resuspended in PBS supplemented with 2% fetal bovine serum (FBS) for immunostaining. Cells were stained with fluorophore-conjugated primary antibodies listed in **Supplementary Table 3** at a concentration of 1 μl per million cells in a 100-μl volume for 30 min on ice. The cells were then washed, resuspended, and filtered in fluorescence-activated cell sorting (FACS) tubes. To identify dead cells, 7-amino-Actinomycin D (BD, Franklin Lakes, NJ, USA) at a final concentration of 0.25 μg/ml was added 10 min before sorting. Data were acquired with a FACSAriaIIIu (BD) and LSRFortessa (BD) and analyzed using FlowJo v10 (Treestar, San Francisco, CA, USA).

### Electron microscopy

Specimens for electron microscopic observation were prepared as described previously with slight modification (46). Briefly, under deep anesthesia, mice were perfused through the left cardiac ventricle with 2.5% glutaraldehyde in 0.1 M phosphate buffer (pH 7.4) followed by physiological saline. Tibialis anterior muscles were then removed and immersed in the same fixative for 2 h. Thereafter, the muscles were cut into about 1-mm^3^ pieces and postfixed with 1% osmium tetroxide in 0.1 M phosphate buffer for 2 h at 4°C. Following dehydration in a graded ethanol series, the samples were embedded in Quetal 812 at 45°C for 1 day and then at 60°C for 2 days. The embedded tissues were then sectioned with an ultramicrotome (Leica EM UC6b/FC6), after which the sections were mounted on copper grids, stained with 8% uranyl acetate and lead citrate, rinsed with deionized water and dried. The sections were then observed and photographed using a JEM-1450 transmission electron microscope (JEOL, Tokyo, Japan).

### RT-PCR analysis

Total RNA was isolated from FACS sorted cells using a RNeasy mini kit (Qiagen) according to the manufacturer’s instructions. Complementary DNA (cDNA) was synthesized using ReverTra Ace qPCR RT Master Mix with genomic DNA (gDNA) Remover (TOYOBO CO., LTD.) All qPCR protocols were performed with StepOne Plus (Applied Biosystems) using a KAPA SYBR FAST ABI Prism qPCR Kit (KAPA BIOSYSTEMS). Values were normalized to the expression of GAPDH and then further normalized to the values in the control samples. Primers are listed in **Supplementary Table 4**.

### RNA-seq

Poly-A mRNA was extracted from total RNA with a NEBnext poly(A) mRNA magnetic isolation module (New England Biolab), after which RNA-seq libraries were prepared using a NEBNext Ultra RNA Library Prep kit for Illumina according to the manufacturer’s protocol (New England Biolab). The libraries were then PCR-amplified for ~12 cycles and sequenced on a Hi-seq 1500 or Novaseq (Illumina). Reads were aligned to the mm10 mouse genome using STAR (47). Expression analysis of the RNA-seq data was performed using HOMER (48). Gene set enrichment analyses were performed using GSEA (49) with rank files generated as previously described (http://genomespot” http://genomespot.blogspot.com/2015/01/how-to-generate-rank-file-from-gene.html) from expression data analyzed using DESeq2 (50). Clustering of GSEA results was performed using EnrichmentMap and AutoAnnotate (26) on Cytoscape (51). After clustering using AutoAnnotate, the gene sets with highly related terms were further collected manually. All RNA-seq data are available in the GEO under the accession number GSE232394.

### Measurement of energy metabolism

The oxygen consumption rate in BMDMs was analyzed using a XE24 Extracellular Flux Analyzer (Seahorse Bioscience). BMDMs were plated in a 24-well Seahorse plate (40,000 cells per well) and incubated overnight in complete medium (DMEM/F12 containing L-glutamine, 10% FBS and 20 ng/mL M-CSF). The next day, the medium was changed to fatty acid oxidation assay medium (111 mM NaCl, 4.7 mM KCl, 1.25 mM CaCl2, 2.0 mM MgSO4, 1.2 mM NaH_2_PO_4_, 2.5 mM glucose, 0.5 mM carnitine and 5 mM Hepes) and incubated for 30 min. Then after pretreatment with etomoxir (40 μM) for 15 min, palmitate-BSA (200 μM palmitate conjugated with 34 μM BSA) or BSA (34 μM) (Seahorse Bioscience) was added, and the assays were initiated. The oxygen consumption rate was measured as pmol of O_2_ consumed per minute. At the same time, the extracellular acidification rate was measured as a change in pH. The wells were sequentially treated with the ATP synthase inhibitor oligomycin (1.0 μM), the chemical uncoupler FCCP (1.5 μM), and the electron transport inhibitor antimycin A/rotenone (0.5 μM each).

### Lipidomics analysis

MS-based lipidomic analysis was performed using our published protocol (52). In brief, for detection of phospholipids, lipids were extracted from cells using the Bligh and Dyer method (53). Electrospray ionization (ESI)-MS analysis was performed using 4000Q-TRAP, a triple quadrupole-linear ion trap hybrid mass spectrometer (Sciex, Framingham, MA, USA) with reverse-phase LC (NexeraX2 system, Shimadzu, Kyoto, Japan). Samples were injected by an autosampler, applied to a Kinetex C18 column (2.1 × 150 mm, 1.7 µm particle, Phenomenex, Torrance, CA, USA) coupled to the ESI-MS, and separated using a step gradient with mobile phase A (acetonitrile/methanol/water = 1:1:1 [v/v/v] containing 5 µM phosphoric acid and 1 mM ammonium formate) and mobile phase B (2-propanol containing 5 µM phosphoric acid and 1 mM ammonium formate) at a flow rate of 0.2 mL/min at 50°C. Lipids were identified based on multiple reaction monitoring (MRM) transition and retention times, and quantification was performed based on the peak area of the MRM transition and on a calibration curve constructed using an authentic standard for each compound. As internal standards, d5-labeled EPA, LPC17:0 and PE14:0-14:0 (500 pmol; Cayman Chemical, Ann Arbor, MI, USA) were added to each sample.

### Statistics

All determinations of statistical significance were done using ANOVA with Tukey’s *post hoc* analysis and/or unpaired Student’s t-tests. P values less than or equal to 0.05 was considered significant.

## Data availability statement

The datasets presented in this study can be found in online repositories. The names of the repository/repositories and accession number(s) can be found below: GEO database under accession number GSE232394.

## Ethics statement

The animal study was approved by Institutional Animal Care Committee at the Nippon Medical School. The study was conducted in accordance with the local legislation and institutional requirements.

## Author contributions

YO, NK, HKo, YN, MA, YT, YM, SM, HKi, TM, HO, IM and carried out the experiments and analyzed the data. YO and IM wrote the manuscript. YO, HS, MM and IM conceived the original idea and supervised the project. All authors contributed to the article and approved the submitted version.
