# Corrigendum: Macrophage SREBP1 regulates skeletal muscle regeneration

**DOI:** 10.3389/fimmu.2024.1382077

**Published:** 2024-04-03

**Authors:** Yumiko Oishi, Hiroyuki Koike, Naoki Kumagami, Yoshimi Nakagawa, Masaya Araki, Yoshitaka Taketomi, Yoshimi Miki, Shigeru Matsuda, Hyeree Kim, Takashi Matsuzaka, Hitoshi Ozawa, Hitoshi Shimano, Makoto Murakami, Ichiro Manabe

**Affiliations:** ^1^ Department of Medical Biochemistry, Graduate School of Medical and Dental Sciences, Tokyo Medical and Dental University, Tokyo, Japan; ^2^ Department of Biochemistry & Molecular Biology, Nippon Medical School, Tokyo, Japan; ^3^ Division of Complex Bioscience Research, Department of Research and Development, Institute of Natural Medicine, University of Toyama, Toyama, Japan; ^4^ Department of Endocrinology and Metabolism, Institute of Medicine, University of Tsukuba, Ibaraki, Japan; ^5^ Laboratory of Microenvironmental Metabolic Health Sciences, Center for Disease Biology and Integrative Medicine, Graduate School of Medicine, The University of Tokyo, Tokyo, Japan; ^6^ Department of Obstetrics and Gynecology, Nippon Medical School, Tokyo, Japan; ^7^ Department of Systems Medicine, Chiba University Graduate School of Medicine, Chiba, Japan; ^8^ Department of Anatomy and Neurobiology, Graduate School of Medicine, Nippon Medical School, Tokyo, Japan

**Keywords:** macrophage, SREBP (sterol regulatory element-binding protein) pathway, EPA - 20:5n-3, skeletal muscle regeneration, fatty acid metabolism

In the published article, there was an error in the author list, and author Hyeree Kim was erroneously excluded. The corrected author list appears below.

Yumiko Oishi, Hiroyuki Koike, Naoki Kumagami, Yoshimi Nakagawa, Masaya Araki, Yoshitaka Taketomi, Yoshimi Miki, Shigeru Matsuda, Hyeree Kim, Takashi Matsuzaka, Hitoshi Ozawa, Hitoshi Shimano, Makoto Murakami, Ichiro Manabe

A correction has also been made to the **Author contributions** section.

The section previously stated:

“YO, NK, HK, YN, MA, YT, YM, SM, TM, HO, IM and carried out the experiments and analyzed the data. YO and IM wrote the manuscript. YO, HS, MM and IM conceived the original idea and supervised the project. All authors contributed to the article and approved the submitted version.”

The corrected **Author contributions** section appears below:

YO, NK, HKo, YN, MA, YT, YM, SM, HKi, TM, HO, IM and carried out the experiments and analyzed the data. YO and IM wrote the manuscript. YO, HS, MM and IM conceived the original idea and supervised the project. All authors contributed to the article and approved the submitted version.

The authors apologize for these errors and state that they do not change the scientific conclusions of the article in any way. The original article has been updated.

