# Supplementary material for: Macrophage SREBP1 regulates skeletal muscle regeneration
Source: Front Immunol. 2024 Jan 8;14:1251784. doi: 10.3389/fimmu.2023.1251784 (PMC10800357; doi:10.3389/fimmu.2023.1251784)
Supplement: Supplementary file 1 [file DataSheet_1.pdf]

Supplemental information

Experimental Method

Satellite cell isolation

Primary satellite cells (SCs) were isolated from the hindlimb muscles of male *Srebf1*$^{-/-}$ and control mice. After excess fat, connective tissue and tendons were removed, the hindlimb muscles were minced and digested in 0.2% collagenase type II (Worthington Biochemical Corp.) for 1 h at 37 °C. Mononuclear cells were stained with PECy7-conjugated anti-CD31, -CD45 and -Ly6A/E as well as fluorescein isothiocyanate-conjugated anti-CD106 antibodies for 30 min on ice. They were then resuspended in phosphate-buffered saline containing 2% fetal bovine serum. SCs were isolated using a fluorescence-activated cell sorting (FACS) Aria III flow cytometer (BD Biosciences). Debris and dead cells were excluded by forward scatter, side scatter and 7-AAD gating. CD45-, CD31- and Ly6A/E-positive cells were removed by gating using PECy7 fluorescence intensity, after which CD106-positive cells were sorted as SCs. Data were collected using FACS Diva software and FlowJo (BD Biosciences). SCs were cultured in GlutaMax DMEM (Life Technologies) supplemented with 20% FBS, 10 ng/mL basic fibroblast growth factor (Cell Signaling Technology, Beverly, MA), 0.2 μg/cm^2 iMatrix-511 silk (Takara bio) and 1% penicillin-streptomycin at 37 °C under 5% CO_2. Myogenic differentiation was induced in GlutaMax DMEM supplemented with 5% horse serum and 1% penicillin-streptomycin on Matrigel-coated plates at 37 °C under 5% CO_2.

Supplemental Figure legends

Supplemental Fig. 1

H-E-stained sections of tibialis anterior muscle collected from wild-type and *Srebf1*$^{-/-}$ mice on the indicated days after cardiotoxin injection. Scale bar: 100μm.

Supplemental Fig. 2

A) Phase contrast images of satellite cells. Muscle satellite cells were purified from hind-limb muscles and cultured in growth medium for the indicated number of days.

B) Immunostaining of undifferentiated satellite cells. Expression of Pax7(green) and MyoD1(red) is shown. Scale bar: 100μm.

C) qPCR analysis of *Myf5*, *Myod1*, *Myog* and *Myh3* mRNA expression. Total RNA from satellite cells grown for 4 days in differentiation medium was collected and subjected to analysis. Data are shown as means ± SD. n=4. ns: not significant. Student's two-tailed t test.

Supplemental Fig. 3

Distribution of muscle fiber cross-sectional area of damaged muscle collected from WT and control *Srebf1*$^{f/f}$ mice 7 days after injury. Data are means ± SD. Shown are the mean numbers of muscle fibers within the indicated area ranges per 1000 myofibers/experimental conditions; one-way ANOVA and Tukey's test for multiple comparisons. *$P < 0.05$.

Supplemental Fig. 4

The UCSC Genome Browser shot showing the ChIP-seq result for SREBP1 in mouse BMDMs. Gene locus of mouse *Naprt, Fahd2a, Pck2, Ldhb and Aldh1b1* are shown.

Supplemental Fig. 5

A-C) Gating strategy. Live cells were subgated based on expression of CD45 and CD11b, and the CD45^{+}CD11b^{+} myeloid cells were subgated based on expression of Ly6G. The Ly6G-population was further subgated based on expression of Ly6C and F4/80 to reveal the Ly6C^{lo} and Ly6C^{hi} populations.

Supplemental Table 1

Fatty acid composition (measured by a gas chromatography)

	Normal chow diet (%)	Fish meal free diet (%)
Decanoic acid (C10:0)	0	0
Lauric acid (C12:0)	0	0
myristic acid (C14:0)	0.5	0.2
Myristoleic acid (C14:1)	0	0
pentadecanoic acid (C15:0)	0	0
pentadecenoic acid (C15:1)	0	0
palmitic acid (C16:0)	16.7	18.3
palmitoleic acid (C16:1)	1.0	0.3
heptadecanoic acid (C17:0)	0.1	0.1
heptadecenoic acid (C17:1)	0	0
stearic acid (C18:0)	2.7	2.9
Oleic acid (C18:1)	27.1	28.8
linoleic acid (C18:2)	44.2	44.3
Linolenic acid (C18:3)	3.4	3.4
Arachidic acid (C20:0)	0.5	0.5
icosenoic acid (C20:1)	0.8	0.5
icosadienoic acid (C20:2)	0	0
Eicosatrienoic acid (C20:3)	0	0
Arachidonic acid (C20:4)	0	0
Eicosapentaenoic acid (C20:5)	1.3	0
Pehenic acid (C22:0)	0.3	0.3
docosadienoic acid (C22:2)	0	0
docosahexaenoic acid (C22:6)	1.1	0
lignoceric acid (C24:0)	0.3	0.4

Supplemental Table 2

Downregulated Hallmark gene sets

Gene sets	NES	FDR q-val
Oxidative phosphorylation	-1.96	0.000
DNA repair	-1.81	0.001
Interferon alpha response	-1.72	0.002
Reactive oxygen species pathway	-1.55	0.016
Interferon gamma response	-1.55	0.013

Upregulated Hallmark gene sets

UV response	2.07	0.001
Mitotic spindle	1.87	0.014
Epithelial mesenchymal transition	1.83	0.013
angiogenesis	1.80	0.013
Protein secretion	1.68	0.028

Downregulated GO Biological processes

Oxidative phosphorylation	-2.26	0.000
Mitochondrial Respiratory chain complex assembly	-2.24	0.000
ATP biosynthetic process	-2.19	0.000
Proton motive force driven ATP synthesis	-2.18	0.000
NADH dehydrogenase complex assembly	-2.14	0.000
Nucleoside triphosphate biosynthetic process	-2.08	0.000
ATP synthesis coupled electron transport	-2.06	0.000
Aerobic electron transport chain	-2.06	0.000
Mitochondrial translation	-2.05	0.000
Aerobic respiration	-2.01	0.000
Electron transport chain	-1.97	0.000
Mitochondrial gene expression	-1.90	0.003

Upregulated GO Biological processes

Collagen fibril organization	2.00	0.737
Cell matrix adhesion	1.96	0.736
Cell substrate adhesion	1.92	0.986
Killing of cells or another organism	1.90	0.838
External encapsulating structure organization	1.88	0.816

Supplemental Table 3

List of antibodies used for flow cytometry and immunofluorescence

Target	Isotype	Conjugate	Clone	Company
Ly-6C	Rat IgG2c	FITC	HK1.4	BioLegend
F4/80	Rat IgG2a	PE	T45-2342	BD
CD45	Rat IgG2b	PE-Cy7	30-F11	BD
Ly-6G	Rat IgG2a	APC-Cy7	1A8	BioLegend
CD11b	Rat IgG2b	Brilliant Violet 510	M1/70	BioLegend
CD11b	Rat IgG2b	PE-Cy7	M1/70	BD
Pax7	Mouse IgG1	Unconjugated	PAX7	Santa cruz
MyoD	Mouse IgG2b	Unconjugated	G-1	Santa cruz
F4/80	Rat IgG2b	Unconjugated	Cl:A3-1	AbD Serotec
CD31	Rat IgG2a	Unconjugated	SZ31	Dianova

Supplemental Table 4

List of primers used in this study

Naprt	Fw: TGCCCTGGCTAGAGTCTGTT
	Rev:TGCTTCTCAGGGTCCTCTGT
Fahd2a	Fw: TCAACACTCCCTAAGACAATGGT
	Rev: TGCATAATTCAAGCCCACACATA
Pck2	Fw: ATGGCTGCTATGTACCTCCC
	Rev:GCGCCACAAAGTCTCGAAC
Ldhb	Fw: CATTGCGTCCGTTGCAGATG
	Rev:GGAGGAACAAGCTCCCGTG
Aldh1b1	Fw: CTCCAGGGCAGGACTACCTC
	Rev:CATGCCACTCGTTGTTGATGA
Cx3cr1	Fw: GAGTATGACGATTCTGCTGAGG
	Rev:CAGACCGAACGTGAAGACGAG
Gapdh	Fw: AATGTGTCCGTCGTGGATCT
	Rev: CATCGAAGGTGGAAGAGTGG

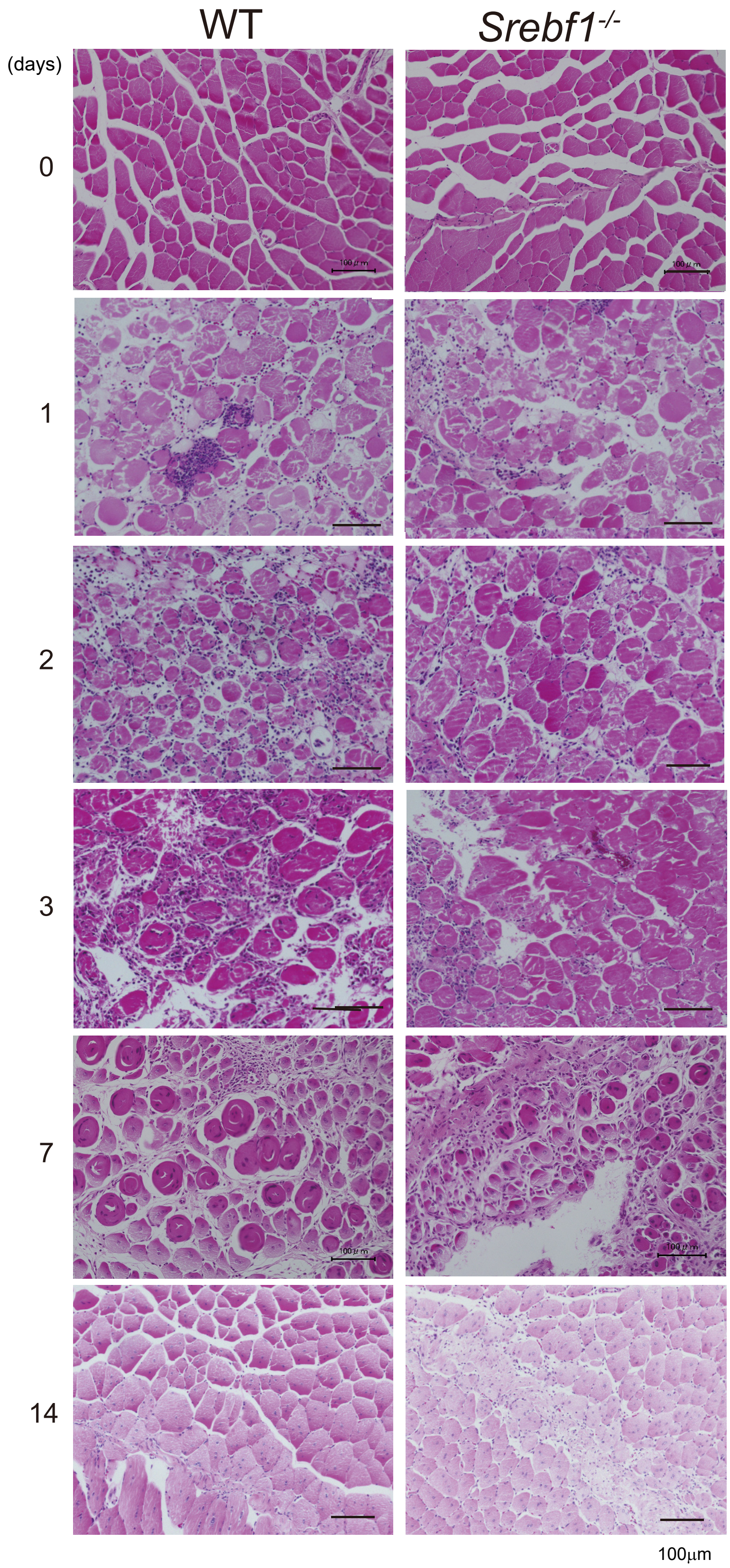
WT
Srebf1$^{-/-}$
(days)
0
1
2
3
7
14
100μm

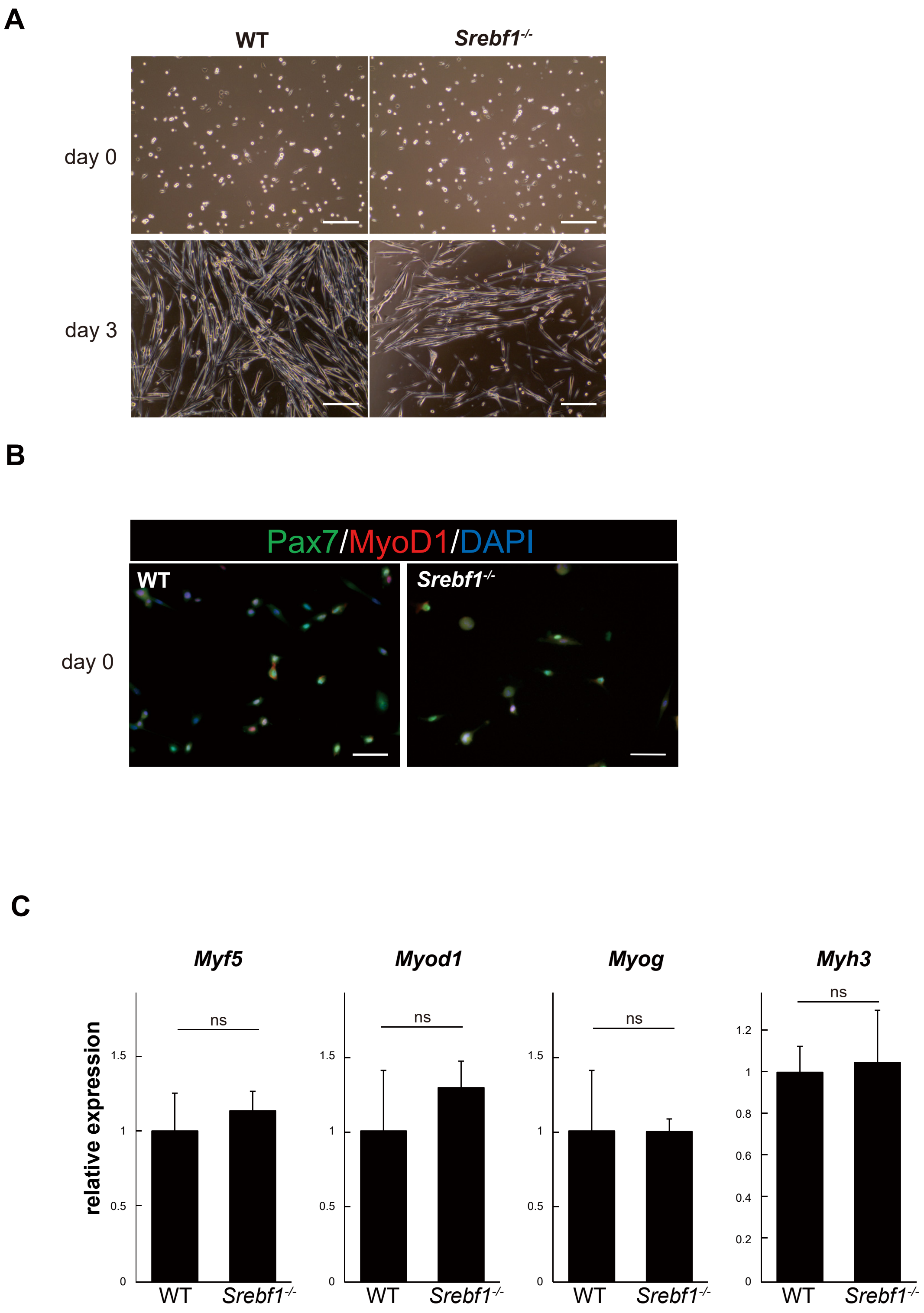
A
WT
Srebf1-/-
day 0
day 3
B
Pax7/MyoD1/DAPI
WT
Srebf1-/-
day 0
C
Myf5
Myod1
Myog
Myh3
relative expression
ns
ns
ns
ns
0
0.5
1
1.5
0
0.2
0.4
0.6
0.8
1
1.2
WT
Srebf1-/-

Suppl. Fig 2

CTX d7

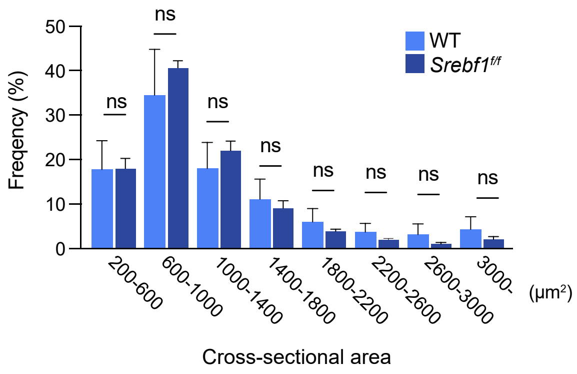

ns
ns
ns
ns
ns
ns
ns
ns
WT
Srebf1f/f
Freqency (%)
50
40
30
20
10
0
200-600
600-1000
1000-1400
1400-1800
1800-2200
2200-2600
2600-3000
3000-
(μm2)
Cross-sectional area

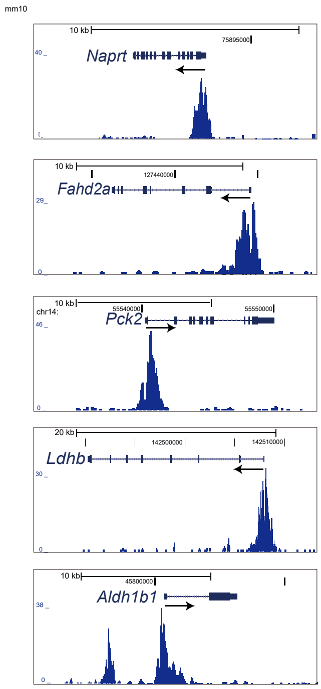
mm10
10 kb
75895000
Naprt
40
10 kb
127440000
Fahd2a
29
0
10 kb
chr14:
55540000
55550000
Pck2
46
0
20 kb
142500000
142510000
Ldhb
30
0
10 kb
45800000
Aldh1b1
38
0

Suppl. Fig. 4

A

Gating strategy related to Fig. 2B

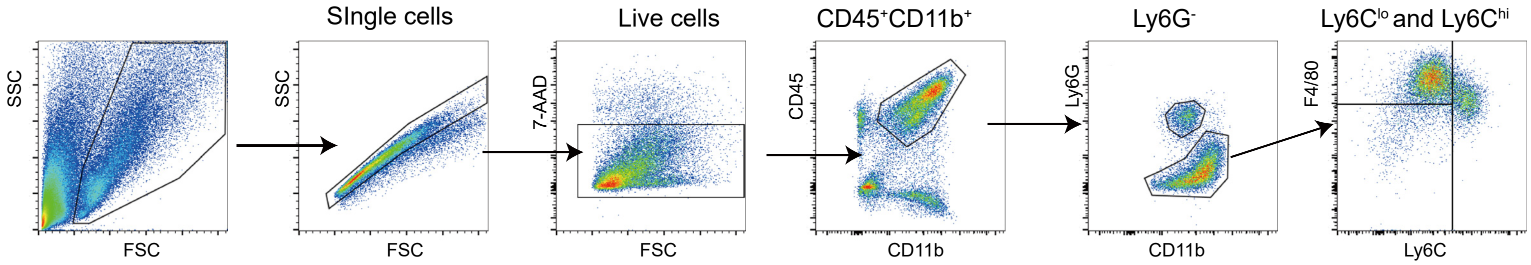

B

Gating strategy related to Fig. 3G

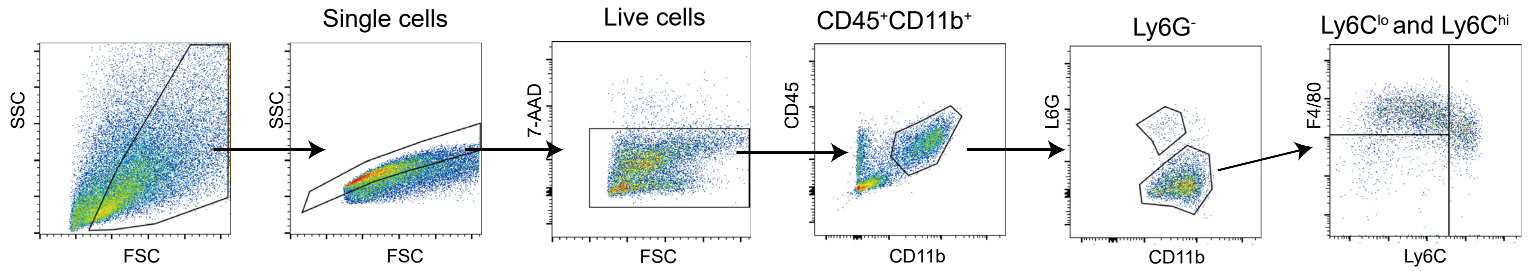

C

Gating strategy related to Fig. 6G

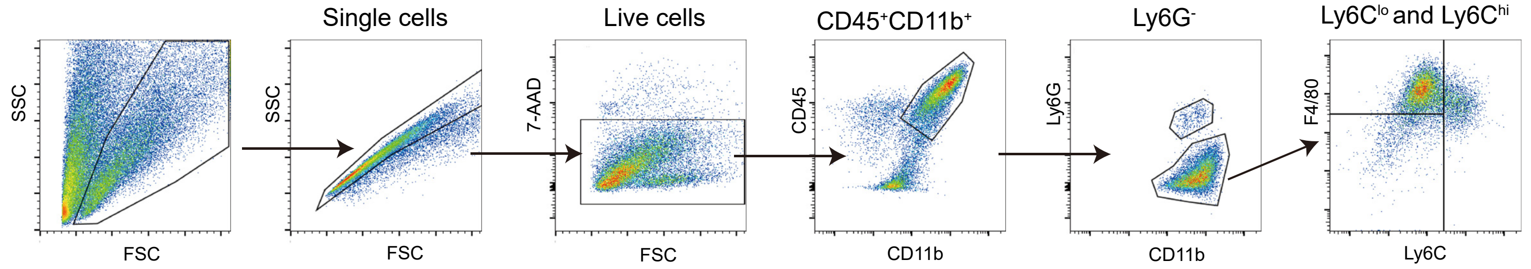

Suppl. Fig.5
